# Effect of Different Parts (Leaf, Stem and Stalk) and Seasons (Summer and Winter) on the Chemical Compositions and Antioxidant Activity of *Moringa oleifera*

**DOI:** 10.3390/ijms12096077

**Published:** 2011-09-19

**Authors:** Ming-Chih Shih, Cheng-Ming Chang, Sue-Ming Kang, Min-Lang Tsai

**Affiliations:** 1 Department of Food, Health and Nutrition Science, Chinese Culture University, 55 Hwa Kang Road, Taipei 11192, Taiwan; E-Mail: smz2@faculty.pccu.edu.tw; 2 Department of Food Science, National Taiwan Ocean University, 2 Pei-Ning Road, Keelung 20224, Taiwan; E-Mails: pcmc@mail.ntou.edu.tw (C.-M.C.); smkang@ems.cku.edu.tw (S.-M.K.)

**Keywords:** *Moringa oleifera*, antioxidative activity, extract, season

## Abstract

*Moringa oleifera*, Lam. (*Moringaceae*) is grown world-wide in the tropics and sub-tropics of Asia and Africa and contains abundant various nutrients. This study describes the effect of different parts (leaf, stem and stalk) and seasons (summer and winter) on the chemical compositions and antioxidant activity of *M. oleifera* grown in Taiwan. The results showed that the winter samples of Moringa had higher ash (except the stalk part), calcium and phenolic compounds (except the leaf part) and stronger antioxidative activity than summer samples. The methanolic extract of Moringa showed strong scavenging effect of DPPH radicals and reducing power. The trend of antioxidative activity as a function of the part of Moringa was: leaf > stem > stalk for samples from both seasons investigated. The Moringa extract showed strong hydrogen peroxide scavenging activity and high Superoxide Dismutase (SOD) activity except the stalk part.

## 1. Introduction

*Moringa oleifera*, Lam. (*Moringaceae*) is grown world-wide in the tropics and sub-tropics of Asia and Africa [1] and is one of the 14 species of genus *Moringa*, which is native to India, Africa, Arabia, Southeast Asia, the Pacific and Caribbean islands, and South America [2]. It is commonly known as horse-radish tree (describing the taste of its roots) or drumstick tree (describing the shape of its pods). The species is drought resistant and tolerate a wide range of soil and rainfall conditions. The flowers and the fruits appear twice each year, and seeds or cuttings can propagate the tree; the latter being preferred [3].

In India and the Philippines, village people use the fresh leaves to prepare fatty foods to increase the shelf life of these foods due to *Moringa* leaves being a good source of natural antioxidants [4]. Recently Anwar and Bhange [5] exploited Moringa as a non-conventional source of oil with significantly high oxidative stability, revealing the presence of natural antioxidants. A report on antioxidant properties of Moringa leaves from different countries that suggested variation has been exploited by Siddhuraju and Becker [4]. Leaves of Moringa have been reported to contain flavonoid pigments such as kaempferol, rhamnetin, isoquercitrin and kaempferitrin [6].

Pal *et al.* [7] reported that the methanol fraction of Moringa leaf extract possesses antiulcer activity against induced gastric lesions in rats. Ghasi *et al.* [8] found that administration of the crude leaf extract of Moringa along with a high-fat diet decreased the high-fat diet-induced increases in serum, liver and kidney cholesterol levels by 14.4%, 6.4% and 11.1%, respectively. Ethanolic extract (50%) of Moringa (whole plant excluding roots) showed anti-cancer activity in mice [9]. The hypotensive activity of ethanolic and aqueous extracts of pods of Moringa was also studied by Faizi *et al.* [10]. They isolated two new compounds along with the known substances methyl *p*-hydroxybenzoate and beta-sitosterol in the study.

Although Moringa is grown world-wide in the tropics and sub-tropics of Asia and Africa, it is still a novel food material in Taiwan. This study describes the effects of different parts (leaf, stem and stalk) and seasons (summer and winter) on the antioxidant potential of Moringa grown in Taiwan.

## 2. Results and Discussion

### 2.1. Proximate Composition of Moringa

Table 1 presents the proximate composition and calcium content of the different parts (leaf, stem and stalk) and harvest seasons (summer and winter) of Moringa. Crude protein content of leaves was 24.42–25.29% suggesting that leaves are a good source of protein. Among the summer samples examined, leaves had the highest content of crude protein, crude fat, and ash. The proximate compositions were similar for summer and winter samples, except crude fat and ash for stalk. Similar results of proximate composition for leaves were reported by Gupta *et al.* [11] and Lowell [12].

The crude protein content (based on wet basis) of Moringa leaves (5.4%) is higher than protein content of alfalfa sprout (3.7%), sweet potato leaves (3.3%), and mung bean sprout (3.1%) being high protein content vegetables usually consumed in Taiwan [13]. According to Hassan and Umar [14], plant food that provides more than 12% (dry basis) of its caloric value from protein is considered a good source of protein. Therefore, Moringa leaves (24.42–25.29% protein) not only meet but even double this requirement.

Moringa leaf also is a good plant source of fat. The crude fat content (1.19–2.77% wet basis) of Moringa leaf is higher compared to reported values (<1.0% wet basis) in most vegetables consumed in Taiwan [13]. Islam *et al.* [15] investigated fifteen different kinds of leafy vegetables available in Bangladesh and found the highest fat content was observed in Moringa leaves.

Ash content was 8.53–11.0% in Moringa leaves which was lower than the investigation of Moringa leaves (15.09% DW) by Lockett *et al.* [16].

Calcium builds healthy bones and teeth and assists in blood-clotting. The seasonal effects on calcium content were different. For winter sample, stalk has highest calcium content while the lowest was stem (Table 1). On the other hand, highest calcium content of Morniga for summer sample was leaf while the lowest was stalk. In Taiwan, the leaves and stems of Moringa are used as vegetables and the stalks are used for soup or stew.

The result showed that the macronutritients may not be affected by season; however, the micronutritients may be affected by season. The mineral content may change during different seasons, which induce significantly different calcium and ash contents between summer and winter. The fact that season’s influence the chemical composition of food is well known. Salvador *et al.* [17] studied the influence of extraction system, production year and area on the chemical compositions of Cornicabra virgin olive oil of five crop seasons and found the crop season was a critical variable.

### 2.2. The Antioxidant Property of Methanolic Extract

Methanol was chosen for extraction in this study because it has wide solubility properties for low molecular and moderately polar substances, including the antioxidant-active phenolic compounds. The extraction ratio, scavenging effect on DPPH radicals and total phenolic compounds of methanolic extracts of Moringa samples from different parts and seasons are shown in Table 2. The trend of scavenging effect on DPPH radicals as a function of the part was: leaf > stem > stalk for both of the seasons’ samples investigated. Meanwhile, the highest content of total phenolic compounds was found in leaves for samples from both seasons investigated. The result also showed that the scavenging effect on DPPH radicals was higher in winter than in summer.

One important mechanism of antioxidation involves the scavenging of hydrogen radicals. DPPH has a hydrogen free radical and shows a characteristic absorption at 517 nm. After encountering the proton-radical scavengers, the purple color of the DPPH solution fades rapidly. The extracts of Moringa are able to reduce the unstable radical DPPH to the yellow-colored diphenylpicrylhydrazine. A dose-response relationship was found in the DPPH radical scavenging activity in this study; the activity increased as the concentration increased for each individual sample. The involvement of free radicals, especially their increased production, appears to be a feature of many diseases including cardiovascular disease and cancer [18]. Phenolic compounds of the extracts are probably involved in their antiradical activity.

The content of total phenolic compounds for the stem and stalk were higher from winter than from summer samples, however, similar contents for the leaves from summer and winter (Table 2). The result was similar to the result of Iqbal and Bhanger [19] who studied the effect of season and production location on antioxidant activity of Moringa leaves grown in Pakistan. This may be due to the fact that Moringa leaves grow in June and mature from December to March and phenolic content is the lowest in newly opened leaves, increasing gradually with the maturity of leaves [19]. In this study, the total phenolic content of the selected samples was determined, however, the phenolic composition of the extracts was not analyzed as it was not within the scope of the present investigation.

Phenolic compounds have an important role in stabilizing lipid oxidation and are associated with antioxidant activity because of their scavenging ability due to their hydroxyl groups [20]. It was determined that there were 181.3–200.0 mg, 71.9–134.4 mg and 68.8–93.8 mg catechin equivalent of phenolic compounds in the 100 g of the leaf, stem and stalk of Moringa, respectively (Table 2). These results indicated that there was no correlation between antioxidant activity and total phenolic content (*p* > 0.05). However, different results were reported on this aspect; some authors found correlation between phenolic content and antioxidant activity [21], whereas the others found no such relationship, since other compounds are responsible for the antioxidant activity [22,23].

Although the phenolic compounds are believed to be the major phytochemicals responsible for antioxidant activity of plant materials [24], Moringa is a rich source of ascorbic acid which is also has the antioxidant activity [25]. To estimate the antioxidant activity contributed from ascorbic acid of Moringa samples, the solvent fraction procedure was performed.

### 2.3. Reducing Power

**Figure 1:**
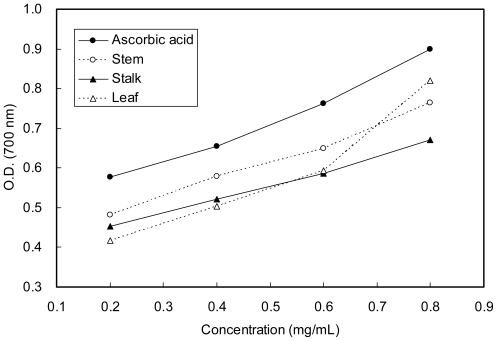
Reducing power of methanolic extracts from different parts of Moringa in comparison with ascorbic acid.

A regular pattern of increase in reducing power as a function of extract concentration of winter’s samples of Moringa was observed (Figure 1). However, there were no significant differences among the three parts of Moringa. Siddhuraju *et al.* [26] showed that the reducing power of bioactive compounds is directly related to ascorbic acid.

Earlier authors [27,28] have observed a direct correlation between antioxidant activity and reducing power of certain plant extracts. The reducing properties are generally associated with the presence of reductones, which have been shown to exert antioxidant action by breaking the free radical chain by donating a hydrogen atom [25]. Reductones are also reported to react with certain precursors of peroxide, thus preventing peroxide formation. From our data on the reducing power of the tested extracts, we suggest that it is likely that reducing power contributed significantly to the observed antioxidant effect.

### 2.4. Hydrogen Peroxide Scavenging Property

**Figure 2:**
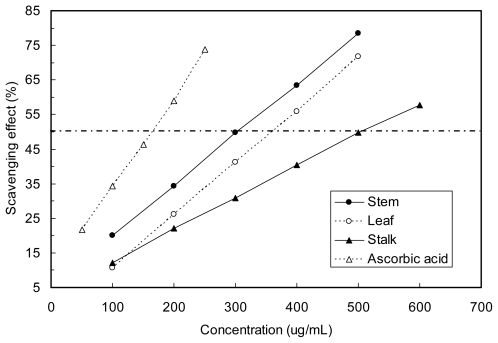
Scavenging effects on hydrogen peroxide from different parts of Moringa.

Hydrogen peroxide scavenging activities of Moringa extracts are shown in Figure 2. All the Moringa samples were capable of scavenging H_2_O_2_ in a concentration dependent manner. As shown in the results, remarkable scavenging effects of the Moringa extracts were observed in hydrogen peroxide scavenging assay of all samples, but the stalk showed the least effect. As shown in Table 3 the Moringa extracts showed an EC_50_ for hydrogen peroxide scavenging effect ranging between 280 and 340 μg/mL, whereas the EC_50_ for ascorbic acid was found to be *ca*. 160 μg/mL. This indicates that all the Moringa extracts tested have substantial H_2_O_2_-scavenging activity, although less than ascorbic acid. Therefore, the reduced amount of hydrogen after this reaction may account for the H_2_O_2_-scavenging effect of the Moringa extracts observed in the present study.

Hydrogen peroxide can be formed *in vivo* by many oxidized enzymes such as superoxide dismutase. Being a non-radical oxygen-containing reactive agent, it can form hydroxyl radical, the most highly reactive oxygen radical known, in the presence of transition metal ions and participate in free-radical reaction [29]. It can cross membranes and may slowly oxidize a number of compounds. Hydrogen peroxide itself is not very reactive, but it can sometimes be toxic to cells because it may give rise to hydroxyl radical in the cells. Thus, removing hydrogen peroxide as well as superoxide anion is very important for protection of food systems.

### 2.5. Superoxide Dismutase (SOD) and Ascorbic Acid

The SOD activity and ascorbic acid content of Moringa extracts are also shown in Table 3. The trend of hydrogen peroxide scavenging activity, SOD activity and ascorbic acid content were similar among the Moringa samples. However, the correlation coefficient between hydrogen peroxide scavenging activity and SOD activity was 0.9410 (*p* < 0.05), and between hydrogen peroxide scavenging activity and ascorbic acid content was −0.7988 (*p* > 0.05). Therefore, SOD may affect the hydrogen peroxide scavenging activity of the Moringa samples.

SOD catalyzes the dismutation of superoxide radical in a broad range of organisms, including plants. The dismutation of superoxide into hydrogen peroxide and oxygen constitute the first line of cellular defense to prevent undesirable biological oxidation by oxygen radical generated during cellular metabolism [30]. In plant cells one of the most important detoxification systems is the water-water cycle (WWC) which operates together with SOD as a mechanism of hydrogen peroxide scavenging in intact chloroplasts [31]. The most important function of this cycle is a rapid, immediate scavenging of O_2_ and H_2_O_2_ at the site of its generation prior to their interaction with the target molecules. Ascorbate peroxidase (APX) uses two molecules of ascorbate to reduce H_2_O_2_ to water, with the concomitant generation of two molecules of monodehydroascorbate (MDHA). MDHA is a radical with a short lifetime, which is reduced directly to ascorbate within the chloroplast at the thylakoid membrane [32].

## 3. Experimental Section

### 3.1. Materials

*Moringa oleifera* was purchased from Spring Autumn Co. in Taichung, Taiwan, and were separately harvested in July 2004, and January 2005. After harvesting, the samples were divided into leaf, stem, and stalk by hand and followed by freeze drying. The freeze dried materials were then ground into powder, and screened through a 20-mesh sieve (aperture, 0.94 mm) and stored at −80 °C.

### 3.2. Chemical Compositions

Crude protein, crude fat and ash of freeze dried Moringa samples were determined according to AOAC Methods 955.04, 920.39 and 930.05 [33], respectively. Calcium was determined as outlined in AOAC Methods 921.01 [33]. Analysis for calcium was carried out using atomic absorption spectroscopy set at 422.7 nm. The burner height was manually adjusted on the instrument to ensure maximum absorption. The composition of all samples had two determinations per replicate.

### 3.3. Preparation of the Methanolic Extracts

Each sample powder (20 g) was extracted with 200 mL of methanol stirred on a stirring plate at room temperature for 24 h. Contents were filtered through #1 filter paper (Whatman Inc., Hillsboro, OR). The filtrate was concentrated to dryness *in vacuo* to obtain methanolic extract. The dried filtrates were weighed to determine the extraction ratio of soluble constituents.

Extraction ratio=Dried filtrate weight (g)/Sample weight (g,dry mass)×100%

The methanolic extract was then stored at −20 °C to determine the scavenging effect on α,α-diphenyl-β-picrylhydrazyl (DPPH) radicals. The methanolic extract was also used to determine the total phenolic compounds, ascorbic acid content and reducing power.

### 3.4. Determination of the Scavenging Effect on DPPH Radicals

The experimental method in the current study was according to Kuo *et al.* [24]. A 400 μM solution of DPPH was prepared in 100% methanol. 50 μL of samples (methanolic extract or various solvent fractions, at final concentration 0–12,000 μg/mL) and 150 μL of DPPH solution were added to each well in a 96-well flat-bottom EIA microtitration plate. After thorough mixing, the solutions were kept in the dark for 90 min. The absorbency of the samples was measured using an Optimax automated microplate reader (Molecular Devices, Toronto, Canada) at 517 nm against methanol without DPPH as the blank reference. Each sample was tested four times and the values were averaged. For the determination of EC_50_ (which is the efficient concentration of antioxidant defined as the 50% of the initial DPPH concentration), each sample was measured at least at five different concentrations in the DPPH test. EC_50_ was obtained by interpolation from linear regression analysis.

### 3.5. Total Phenolic Compounds

Total phenolic compounds were measured with Folin-Ciocalteu reagent using catechin as a standard [34]. A 5 mL of Folin-Ciocalteu reagent (diluted tenfold in distilled water), 2 mL of 200 g/L sodium bicarbonate, and 2 mL of distilled water were added to 1 mL of the raw methanolic extract of Moringa samples. After 1 h at 20 °C, the absorbance at 755 nm was read.

### 3.6. Reducing Power

Reducing power was determined following the method reported by Oyaizu [35]. Each 5 mL of methanolic extracts (0.2, 0.4, 0.6 and 0.8 mg/mL) was mixed with phosphate buffer (5.0 mL, 2.0 M, pH 6.6) and 1% potassium ferricyanide (5 mL), and the mixtures were incubated at 50 °C for 20 min. A 5mL of 10% trichloroacetic acid was added and the mixture was centrifuged at 650× g for 10 min. The upper layer of the solution (5 mL) was mixed with distilled water (5 mL) and 0.1% ferric chloride (1 mL), and absorbance was measured at 700 nm. The experiment was conducted in triplicate and results were averaged.

### 3.7. Hydrogen Peroxide Scavenging Property

Hydrogen peroxides were measured using the horseradish peroxidase assay [36]. A 1 mL of Moringa extract sample was first mixed with 400 μL of 4 mM H_2_O_2_ solution and allowed to incubate for 20 min at room temperature. Then 600 μL of phenol red solution (7.5 mM phenol red and 500 μg/mL horseradish peroxidase in 100 mM phosphate buffer) was added to the reaction mixture. After 10 min, the absorbance was measured at 610 nm. Sample absorbances were converted to mM H_2_O_2_ by interpolating from a standard curve.

Scavenging effect (%)=1-(Absorbance of sample/Absorbance of control)×100%

### 3.8. Superoxide Dismutase (SOD)

Determination of SOD activity was performed by using a kit (Ransod, Randox Labs. cat. no. SD 125, Antrim, UK) based on the method developed by McCord and Fridovich [37]. A 5 μL of Moringa extract sample was added concomitantly with the main reagent (170 μL) to the cuvette. Absorbance was monitored at 500 nm for 150 s after addition of xanthine oxidase (25 μL plus 10 μL of H_2_O) as start reagent. Read initial absorbance (A1) after 30 s and start timer simultaneously. Final absorbance (A2) was read after 3 min.

$$\begin{array}{c} (\text{A}2-\text{A}1)/3=\Delta \text{A}/\text{min}\;\text{of standard or sample}\\ \text{Sample diluents rate }(\text{S}1\;\text{rate})=\text{rate of}\;\text{inhibited reaction}=100\% \end{array}$$

All standard rates and sample diluents rates must be converted into percentages of the sample diluents rate, and subtracted from 100% to give a percentage inhibition.

$$100-[(\Delta {\text{A}}_{\text{sample}/\text{min}}\times 100)/(\Delta {\text{A}}_{\text{s}1/\text{min}})]=\%\;\text{inhibition}$$

Each sample was tested four times and the values were averaged. For the determination of EC_50_, each sample was measured at least at five different concentrations in the test. EC_50_ was obtained by interpolation from linear regression analysis.

### 3.9. Ascorbic Acid

Ascorbic acid was measured by a Merck, RQ flex plus (Darmstadt, Germany) spectrophotometer [38]. A 1 mL of Moringa extract sample was diluted to properly concentration before measurement. An ascorbic acid test strip (25–450 mg/L) was immersed in the liquid for 5 s before leaving a further 55 s and reading the change in color intensity using a RQ Flex plus spectrophotometer.

### 3.10. Statistical Analysis

Data were analyzed by analysis of variance (ANOVA) using general linear model. Duncan’s multiple range test was used to determine the differences among samples. Significant levels were defined as probabilities of 0.05 or less. All processing treatments were done in triplicate.

## 4. Conclusions

As a conclusion, the winter samples of Moringa had higher ash (except the stalk part), calcium and phenolic compounds (except the leaf part) and stronger antioxidative activity than summer samples. The methanolic extract of Moringa showed strong scavenging effect of DPPH radical and reducing power. The trend of antioxidative activity as a function of the parts of Moringa was: leaf > stem > stalk for samples from both seasons investigated. Meanwhile, the Moringa extract of Moringa showed stronger hydrogen peroxide scavenging activity and higher SOD activity except the stalk part. The results of this study showed that the methanol extracts of Moringa can be used as an easily accessible source of natural antioxidants in the food and pharmaceutical industries. However, the phenolic compounds or other components responsible for the antioxidant activity of methanol extracts of Moringa are still unknown. Therefore, it is suggested that further work could be performed on the isolation and identification of the antioxidant components in Moringa.

## Figures and Tables

**Table 1:** Proximate composition and calcium content of Moringa from different parts and seasons.

Part	Season	Crude protein(%db) *	Crude fat(%db)	Ash (%db)	NFE (%db) **	Ca (mg/100 gdb)
Leaf	Summer	25.29 ^A^	5.75 ^A^	8.53 ^B^	60.43	870 ^D^
	Winter	24.42 ^A^	5.37 ^A^	11.00 ^A^	59.21	1862 ^B^

Stem	Summer	12.77 ^B^	2.00 ^B^	6.65 ^C^	78.58	780 ^E^
	Winter	9.56 ^B^	1.98 ^B^	8.41 ^B^	80.05	1562 ^C^

Stalk	Summer	5.29 ^C^	1.38 ^B^	6.48 ^C^	86.85	761 ^F^
	Winter	7.07 ^BC^	1.00 ^C^	2.91 ^D^	89.02	2247 ^A^

* dry basis (g/g, %).

** NFE = Nitrogen free extract = 100 − crude protein − crude fat − ash. Within a column followed by the same letter (^A–F^) don’t differ at the 5% level by Duncan’s multiple range test.

**Table 2:** The effects of methanolic extracts of Moringa on the scavenging effect of DPPH radicals and total phenolics from different parts and seasons.

Part	Summer			Winter		

	Recovery (%)	DPPH (EC_50_) (μg/mL)	Phenolics (mg/100g db)	Recovery (%)	DPPH (EC_50_) (μg/mL)	Phenolics (mg/100 g db)
Leaf	51.95	387 ^C^	200.0 ^A^	81.72	200 ^C^	181.3 ^A^
Stem	60.86	1116 ^B^	71.9 ^B^	77.50	316 ^B^	134.4 ^B^
Stalk	30.25	1874 ^A^	68.8 ^B^	85.38	624 ^A^	93.8 ^C^

Within a column followed by the same letter (^A–C^) don’t differ at the 5% level by Duncan’s multiple range test.

**Table 3:** Antioxidant activity of different parts of Moringa extract.

Part	H_2_O_2_ scavenging effect (EC_50_) (μg/mL)	Superoxide Dismutase (SOD) (EC_50_) (μg/mL)	Ascorbic acid (mg/100 g db)
Leaf	340 ^B^	2.00 ^B^	780 ^A^
Stem	280 ^B^	7.92 ^B^	590 ^B^
Stalk	530 ^A^	50.00 ^A^	310 ^C^

Within a column followed by the same letter (^A–C^) don’t differ at the 5% level by Duncan’s multiple range test.
